# Comparative cardiovascular and renal effectiveness of empagliflozin and dapagliflozin: Scandinavian cohort study

**DOI:** 10.1093/ehjcvp/pvae045

**Published:** 2024-06-25

**Authors:** Arvid Engström, Jonas Söderling, Anders Hviid, Björn Eliasson, Soffia Gudbjörnsdottir, Viktor Wintzell, Kristian Hveem, Christian Jonasson, Mads Melbye, Björn Pasternak, Peter Ueda

**Affiliations:** Clinical Epidemiology Division, Department of Medicine, Karolinska Institutet, Solna, Stockholm 171 77, Sweden; Clinical Epidemiology Division, Department of Medicine, Karolinska Institutet, Solna, Stockholm 171 77, Sweden; Department of Epidemiology Research, Statens Serum Institut, DK-2300 Copenhagens, Denmark; Pharmacovigilance Research Center, Department of Drug Design and Pharmacology, Faculty of Health and Medical Sciences, University of Copenhagen, DK-1165 Copenhagen, Denmark; Department of Molecular and Clinical Medicine, Institute of Medicine, University of Gothenburg, Gothenburg 405 30, Sweden; Department of Molecular and Clinical Medicine, Institute of Medicine, University of Gothenburg, Gothenburg 405 30, Sweden; The Swedish National Diabetes Register, Vastra Gotalandsregionen, Gothenburg 413 45, Sweden; Clinical Epidemiology Division, Department of Medicine, Karolinska Institutet, Solna, Stockholm 171 77, Sweden; HUNT Center for Molecular and Clinical Epidemiology, Department of Public Health and Nursing, Faculty of Medicine and Health Science, NTNU—Norwegian University of Science and Technology, Trondheim NO-7491, Norway; HUNT Research Center, Faculty of Medicine, NTNU—Norwegian University of Science and Technology, Levanger 7600, Norway; HUNT Center for Molecular and Clinical Epidemiology, Department of Public Health and Nursing, Faculty of Medicine and Health Science, NTNU—Norwegian University of Science and Technology, Trondheim NO-7491, Norway; HUNT Research Center, Faculty of Medicine, NTNU—Norwegian University of Science and Technology, Levanger 7600, Norway; Department of Clinical Medicine, University of Copenhagen, DK-1165 Copenhagen, Denmark; Department of Genetics, Stanford University School of Medicine, Stanford, CA 94305-5176, USA; HUNT Center for Molecular and Clinical Epidemiology, Department of Public Health and Nursing, Faculty of Medicine and Health Science, NTNU—Norwegian University of Science and Technology, Trondheim NO-7491, Norway; Danish Cancer Institute, DK-2100 Copenhagen, Denmark; Clinical Epidemiology Division, Department of Medicine, Karolinska Institutet, Solna, Stockholm 171 77, Sweden; Department of Epidemiology Research, Statens Serum Institut, DK-2300 Copenhagens, Denmark; Clinical Epidemiology Division, Department of Medicine, Karolinska Institutet, Solna, Stockholm 171 77, Sweden

**Keywords:** SGLT2 inhibitors, Comparative effectiveness, Dapagliflozin, Empagliflozin, Heart failure, Chronic kidney disease, Diabetic ketoacidosis

## Abstract

**Aims:**

To assess the comparative cardiovascular and renal effectiveness and safety of empagliflozin vs. dapagliflozin among patients with type 2 diabetes in routine clinical practice.

**Methods and results:**

Cohort study using data from nationwide registers in Sweden, Denmark, and Norway, from June 2014 to June 2021 included 141 065 new users of empagliflozin and 58 306 new users of dapagliflozin. Coprimary outcomes were major cardiovascular events (myocardial infarction, stroke, and cardiovascular death), heart failure (hospitalization or death because of heart failure) and serious renal events (renal replacement therapy, hospitalization for renal events, and death from renal causes). Secondary outcomes were the individual components of the primary outcomes, any cause death, and diabetic ketoacidosis. Use of empagliflozin vs. dapagliflozin was associated with similar risk of major cardiovascular events [adjusted incidence rate: 15.9 vs. 15.8 events per 1000 person-years; HR 1.02, (95% confidence interval 0.97–1.08)], heart failure [6.5 vs. 6.3 events per 1000 person-years; HR 1.05 (0.97–1.14)] and serious renal events [3.7 vs. 4.1 events per 1000 person-years; HR 0.97 (0.87–1.07)]. In secondary outcome analyses, the HRs for use of empagliflozin vs. dapagliflozin were 1.00 (0.93–1.07) for myocardial infarction, 1.03 (0.95–1.12) for stroke, 1.01 (0.92–1.13) for cardiovascular death, 1.06 (1.00–1.11) for any cause death, 0.77 (0.60–0.99) for renal replacement therapy, 1.20 (0.75–1.93) for renal death, 1.01 (0.90–1.12) for hospitalization for renal events and 1.12 (0.94–1.33) for diabetic ketoacidosis.

**Conclusion:**

Use of empagliflozin and dapagliflozin was associated with similar risk of cardiovascular and renal outcomes, mortality, and diabetic ketoacidosis.

## Introduction

Sodium-glucose cotransporter-2 (SGLT2) inhibitors have a central role in cardiovascular and renal risk reduction among patients with type 2 diabetes. Empagliflozin and dapagliflozin are the most frequently prescribed SGLT2 inhibitors worldwide.^1^ However, much uncertainty remains regarding their comparative effectiveness and safety, with implications for guideline recommendations and use of the drugs. For example, the US Food and Drug Administration has approved reduction of cardiovascular death as an indication for empagliflozin in type 2 diabetes, while the corresponding indication for dapagliflozin is reduction of heart failure hospitalization. For patients with high risk or established atherosclerotic cardiovascular disease, the 2022 EASD/ADA consensus report recommends using an SGLT2 inhibitor with proven cardiovascular benefit and highlights empagliflozin, but not dapagliflozin, as a drug with beneficial effects on major adverse cardiovascular events.^2^

The differential approval of indications and guideline recommendations for empagliflozin vs. dapagliflozin are based on interpretations of the large cardiovascular outcome trials. In the EMPA-REG OUTCOME, randomization to empagliflozin vs. placebo in patients with type 2 diabetes led to significant reductions in major adverse cardiovascular events, although this was driven by cardiovascular death. Empagliflozin also reduced risk of hospitalization for heart failure as well as any cause death.^3^ In contrast, DECLARE-TIMI 58 found that randomization to dapagliflozin vs. placebo reduced risk of the composite outcome of cardiovascular death or hospitalization for heart failure; the effect was driven by a reduction in hospitalization for heart failure, while there was no statistically significant reduction in cardiovascular death. There were also no statistically significant reductions in any cause of death or major adverse cardiovascular events.^4^ For renal outcomes, both empagliflozin and dapagliflozin showed protective effects and subsequent dedicated renal outcome trials, including DAPA-CKD^5^ and EMPA-KIDNEY,^6^ confirmed renoprotective effects for both drugs in patients with and without established chronic kidney disease and with and without diabetes.

Differences in outcome definitions and the proportion of patients with established cardiovascular disease between the cardiovascular outcome trials of empagliflozin and dapagliflozin could potentially explain the partly inconsistent results of EMPA-REG OUTCOME and DECLARE-TIMI 58. There are also pharmacological differences, including receptor selectivity, between the drugs that could translate into differential effects on outcomes.^7^ Given the lack of head-to-head trials, it remains unclear whether empagliflozin and dapagliflozin differently affect the risk of cardiovascular and renal outcomes.

SGLT2 inhibitors are associated with increased risk for diabetic ketoacidosis.^3,4,8–10^ Clinical trials and observational studies suggest a class effect, but the previous analyses have been limited by small numbers of events and whether there is difference in risk for diabetic ketoacidosis between empagliflozin and dapagliflozin is uncertain.

The objective of this study was to compare the cardiovascular and renal effectiveness and safety of empagliflozin vs. dapagliflozin using nationwide data from routine clinical practice in Sweden, Denmark, and Norway.

## Methods

### Data sources and study design

We conducted a cohort study with an active-comparator new-user design, using individual-level data from national registers in Sweden, Denmark, and Norway. We used data from population registers (vital status, demographics), Statistics Denmark/Sweden (socioeconomic variables), patient registers (comorbidities, outcomes), prescribed drug registers (study drugs, co-medications), the Swedish National Diabetes Register [glycated haemoglobin level, blood pressure, albuminuria, estimated glomerular filtration rate (eGFR), body mass index, and smoking], and the Danish Register of Laboratory Results for Research (glycated haemoglobin, albuminuria, and eGFR; details are provided in the Supplementary material online, Appendix).

### Study population

We included patients, aged 35–84 years, who were new users of empagliflozin or dapagliflozin between 1 June 2014 and 30 June 2021 in Sweden and Denmark and between 1 June 2014 and 31 December 2018 in Norway. New use was defined as no use of a SGLT2 inhibitor at any time before cohort entry. The recommended starting dose for both empagliflozin and dapagliflozin was 10 mg during the study period.^11,12^ The anatomic therapeutic chemical codes for the study drugs are provided in Supplementary material online, *Table S1*. The date of filling the first prescription constituted cohort entry.

We excluded patients who did not use any glucose-lowering drug within 6 months before cohort entry and who also had a history of chronic heart failure or chronic kidney disease at any time before cohort entry. Hence, patients without type 2 diabetes who were prescribed SGLT2 inhibitors for heart failure or chronic kidney disease were excluded. Further exclusion criteria were history of dialysis or renal transplantation, end stage illness, drug misuse, severe pancreatic disorders, neither use of any prescription drug nor any specialist care contact in the previous year, and hospital admission for any reason within 30 days before cohort entry (Supplementary material online, *Table S2*).

### Outcomes

The study had three coprimary outcomes: (1) major cardiovascular events (a composite of myocardial infarction, stroke, and cardiovascular death); (2) heart failure (hospital admission for heart failure or death due to heart failure); and (3) serious renal events (a composite of renal replacement therapy, death from renal causes, and hospitalization for renal events). Renal replacement therapy is defined as dialysis or renal transplantation. Hospitalization for renal events was based on events consistent with serious renal disease including chronic kidney disease, acute kidney injury, and diabetic nephropathy and was considered a renal analogue to the frequently used outcome of hospital admission for heart failure. Secondary outcomes were the individual components of the coprimary outcomes and any cause death. We also analysed a serious adverse event outcome of concern with empagliflozin and dapagliflozin: diabetic ketoacidosis. The International Classification of Diseases (version 10) codes and procedure codes used to define the outcomes are shown in Supplementary material online, *Tables S3* and *S4*.

### Follow up

Patients were followed from cohort entry (date of first prescription) until outcome event, death, emigration, 5 years of follow-up, or end of study period. Patients were defined as exposed to the study drug from cohort entry throughout follow-up, analogous to an intention-to-treat design in a clinical trial. Each coprimary and secondary outcome was analysed separately.

### Statistical analyses

We used inverse probability of treatment weighting with stabilized weights to adjust for confounding.^13^ A logistic regression model was used to estimate the propensity score, defined as the probability of initiating empagliflozin vs. dapagliflozin conditional on 68 baseline covariates. The covariates included sociodemographic characteristics, diabetes complications, co-morbidities, antidiabetic medications, non-diabetes medications and measures of burden of co-morbidities, frailty, and healthcare utilization (Supplementary material online, *Table S5*). The propensity scores were estimated in each country separately and patients outside the overlapping regions of the propensity score distribution were excluded. Analyses were performed on a pooled dataset from the three countries. The covariate balance after weighting was assessed with standardized differences; differences of less than 10% were considered indicative of good balance. Missing data on education (<3%) was handled with the use of missing categories.^14^ There was no missing data for the other variables included in the propensity score.

A Cox proportional hazards regression model with time since start of treatment as the time scale was used to estimate hazard ratios. The absolute rate difference was calculated using a Poisson model with identity link.^15^ Ninety-five percent confidence intervals (CIs) that did not overlap 1 were considered statistically significant. We described the cumulative incidence using Kaplan-Meier curves. Risk differences for the coprimary outcomes at 1 year, 3 years, and 5 years after cohort entry were estimated by the adjusted Kaplan-Meier estimator, with 95% CIs estimated using bootstrapping.

We conducted prespecified subgroup analyses of coprimary outcomes and the secondary outcomes cardiovascular death and any cause death by age group (35–64 years and ≥65–84 years), sex, history of major cardiovascular disease, history of heart failure and history of chronic kidney disease (Supplementary material online, *Table S6*). A separate propensity score was estimated within each subgroup. Effect modification by subgroup status was examined with an interaction term between treatment status and subgroup; in these analyses, *P*-values of <0.05 were considered statistically significant. We also analysed the coprimary outcomes by country.

We conducted prespecified sensitivity analyses to assess the robustness of the findings. First, in the Swedish and Danish parts of the cohort, we conducted analyses of the coprimary outcomes in which we expanded the propensity score to include additional variables providing information about disease severity and comorbidities: glycated haemoglobin level, blood pressure, albuminuria, eGFR, body mass index, and smoking in Sweden and glycated haemoglobin level, albuminuria, and eGFR in Denmark (Supplementary material online, *Table S7*). Given the proportion of missing values for the additional variables (Supplementary material online, *Table S7*), multiple imputation (fully conditional specification imputation) with 10 imputed datasets was used.^16^ Imputation was based on all variables included in the propensity score, the additional variables, and the outcome variable. Second, we performed analyses of the coprimary outcomes and the secondary outcomes of cardiovascular death and any cause death in which we used an as-treated exposure definition. Patients were considered exposed and remaining on treatment as long as prescriptions were refilled within the estimated duration of the most recent prescription. A 30-day grace period was used.

The study was approved by the Regional Ethics Committee in Stockholm, Sweden, and the Regional Committee for Medical and Health Research Ethics, Norway. In Denmark, ethics approval is not required for register-based research.

## Results

**Figure 1:**
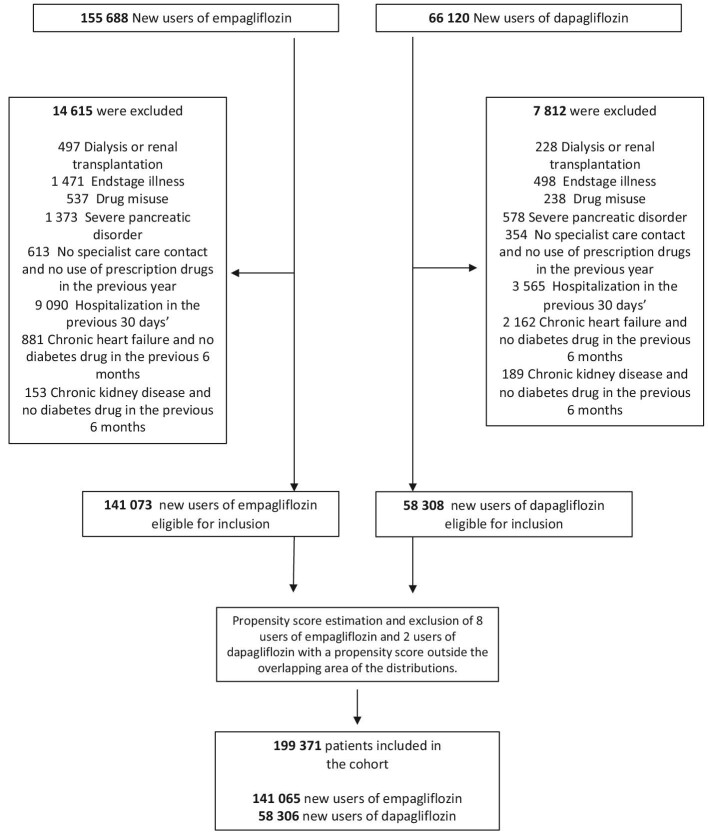
Flow chart of patient inclusion in the study cohort, Sweden, Denmark, and Norway.

The cohort included 141 065 new users of empagliflozin and 58 306 new users of dapagliflozin (*Figure 1*). Population characteristics before and after propensity score weighting are shown in *Table 1*. All covariates in the two groups were well-balanced after weighting. The mean age of the study population was 63 years and 36% were female. The median (IQR) follow-up time was 2.0 (0.9–3.2) years for users of empagliflozin and 3.0 (1.3–4.5) for users of dapagliflozin.

**Table 1:** Patient characteristics at cohort entry for users of empagliflozin and dapagliflozin before and after propensity score weighting.

	Unweighted, *n* (%)			Propensity score weighted, %		
	Empagliflozin (*N* = 141 065)	Dapagliflozin (*N* = 58 306)	Standardized difference (%)	Empagliflozin	Dapagliflozin	Standardized difference (%)
Male	90 688 (64.3)	36 203 (62.1)	4.6	63.8	63.2	1.3
Age, mean (SD) in years	63.2 (10.5)	61.8 (10.6)	-	63.0 (10.5)	62.4 (10.6)	
Age group in years						
35–39	2523 (1.8)	1324 (2.3)	3.4	1.9	2.1	1.4
40–44	5038 (3.6)	2624 (4.5)	4.7	3.7	4.1	2.1
45–49	9395 (6.7)	4782 (8.2)	5.9	6.9	7.6	2.7
50–54	15 452 (11.0)	7324 (12.6)	5.0	11.2	11.9	2.2
55–59	20 019 (14.2)	8848 (15.2)	2.8	14.3	14.8	1.4
60–64	23 052 (16.3)	9586 (16.4)	0.3	16.4	16.3	0.2
65–69	24 021 (17.0)	9517 (16.3)	1.9	17.0	16.4	1.5
70–74	22 501 (16.0)	7924 (13.6)	6.7	15.5	14.7	2.3
75–79	13 668 (9.7)	4477 (7.7)	7.2	9.3	8.5	2.8
80–84	5396 (3.8)	1900 (3.3)	3.1	3.7	3.5	1.2
Place of birth						
Scandinavia	112 780 (79.9)	47 659 (81.7)	4.6	80.1	81.4	3.3
Rest of Europe	10 922 (7.7)	4036 (6.9)	3.1	7.7	7.0	2.5
Outside Europe	17 363 (12.3)	6611 (11.3)	3.0	12.2	11.6	1.9
Civil status						
Married/living with partner	79 471 (56.3)	33 876 (58.1)	3.6	56.2	58.3	4.4
Single	61 594 (43.7)	24 430 (41.9)	3.6	43.8	41.7	4.4
Education						
Primary-/secondary school vocational training	97 959 (77.2)	34 252 (78.2)	2.3	77.3	78.0	1.7
Short tertiary education	10 632 (8.4)	2933 (6.7)	6.4	8.4	6.7	6.1
Medium or long tertiary education	15 445 (12.2)	5527 (12.6)	1.3	12.1	12.7	1.8
Missing	2846 (2.2)	1100 (2.5)		2.2	2.5	
Year of cohort entry^a^						
2014–2015	3736 (2.6)	16 358 (28.0)	-	2.6	27.7	-
2016–2017	33 505 (23.8)	16 496 (28.3)	-	23.8	28.0	-
2018–2019	57 146 (40.5)	13 168 (22.6)	-	40.6	22.4	-
2020–2021	46 678 (33.1)	12 284 (21.1)	-	33.0	21.8	-
Comorbidities						
Acute coronary syndrome	13 846 (9.8)	3658 (6.3)	13.1	9.1	7.8	4.7
Other ischaemic heart disease	27 632 (19.6)	8553 (14.7)	13.1	18.4	17.4	2.8
Heart failure/cardiomyopathy	9486 (6.7)	3375 (5.8)	3.9	6.7	6.1	2.4
Valve disorders	4150 (2.9)	1410 (2.4)	3.3	2.8	2.9	0.8
Stroke	6336 (4.5)	2060 (3.5)	4.9	4.3	4.0	1.8
Other cerebrovascular disease	6900 (4.9)	2331 (4.0)	4.3	4.7	4.4	1.6
Atrial fibrillation	12 264 (8.7)	4146 (7.1)	5.9	8.5	7.8	2.5
Other arrhythmia	6712 (4.8)	2375 (4.1)	3.3	4.6	4.5	0.4
Arterial disease (including amputation)	7936 (5.6)	3174 (5.4)	0.8	5.4	6.0	2.5
Chronic kidney disease	4622 (3.3)	2139 (3.7)	2.2	3.3	3.7	1.9
Other renal disease	10 270 (7.3)	3526 (6.0)	4.9	7.2	6.4	3.0
Diabetes complications	35 911 (25.5)	14 757 (25.3)	0.3	25.1	26.2	2.5
COPD	5255 (3.7)	2141 (3.7)	0.3	3.6	3.8	1.0
Other lung disease	10 068 (7.1)	3758 (6.4)	2.8	7.0	6.8	1.0
Venous thromboembolism	3667 (2.6)	1269 (2.2)	2.8	2.6	2.3	1.5
Cancer (excl non-melanoma skin cancer)	10 953 (7.8)	3935 (6.7)	3.9	7.6	7.2	1.6
Liver disease	3255 (2.3)	1204 (2.1)	1.7	2.3	2.2	0.7
Rheumatic disease	4382 (3.1)	1669 (2.9)	1.4	3.0	3.0	0.3
Psychiatric disorder	13 282 (9.4)	4665 (8.0)	5.0	9.4	8.1	4.8
Alcohol related disorders	2589 (1.8)	928 (1.6)	1.9	1.8	1.6	2.0
Coronary revascularization in previous year	2939 (2.1)	492 (0.8)	10.4	1.9	1.4	4.0
Other cardiac surgery/invasive cardiac procedure in previous year	1454 (1.0)	399 (0.7)	3.8	0.9	1.0	0.2
Fracture in previous year	2279 (1.6)	956 (1.6)	0.2	1.6	1.6	0.3
Health care utilization in previous year						
Hospitalization due to cardiovascular causes	7770 (5.5)	2454 (4.2)	6.0	5.2	5.0	0.9
Hospitalization due to heart failure	975 (0.7)	501 (0.9)	1.9	0.7	0.7	0.1
Hospitalization due to renal causes	1529 (1.1)	700 (1.2)	1.1	1.1	1.2	0.7
Hospitalization due to type 2 diabetes	1332 (0.9)	586 (1.0)	0.6	0.9	1.1	2.0
Hospitalization due to other causes	17 578 (12.5)	7330 (12.6)	0.3	12.2	13.2	3.0
Outpatient contact due to cardiovascular causes	16 147 (11.5)	5493 (9.4)	6.6	10.9	10.8	0.5
Outpatient contact due to heart failure	2464 (1.7)	1205 (2.1)	2.3	1.9	1.8	0.9
Outpatient contact due to renal causes	3281 (2.3)	1377 (2.4)	0.2	2.3	2.4	0.5
Outpatient contact due to type 2 diabetes	22 424 (15.9)	10 835 (18.6)	7.1	15.0	20.8	15.0
Outpatient contact due other causes	74 310 (52.7)	30 631 (52.5)	0.3	52.4	53.4	2.1
Diabetes drugs in previous 6 months						
No diabetes drug	7061 (5.0)	3179 (5.5)	2.0	5.1	5.1	0.1
Metformin	116 732 (82.8)	47 899 (82.2)	1.6	82.2	83.5	3.4
Sulfonylureas	21 130 (15.0)	11 013 (18.9)	10.4	15.6	17.3	4.7
GLP1 receptor agonists	23 270 (16.5)	9364 (16.1)	1.2	16.4	16.5	0.3
DPP4 inhibitors	37 774 (26.8)	20 060 (34.4)	16.6	28.0	31.6	7.9
Insulin	36 535 (25.9)	12 574 (21.6)	10.2	25.6	22.2	8.0
Other antidiabetics	4692 (3.3)	1486 (2.5)	4.6	3.4	2.4	6.2
Time since first diabetes drug						
<1 y	14 214 (10.1)	5761 (9.9)	0.7	9.9	10.1	0.7
1–<3 y	16 529 (11.7)	6596 (11.3)	1.3	11.6	11.4	0.6
3–<5 y	16 735 (11.9)	6945 (11.9)	0.1	11.9	11.8	0.5
5–<7 y	16 896 (12.0)	7495 (12.9)	2.7	12.2	12.3	0.3
≥7 y	76 691 (54.4)	31 509 (54.0)	0.7	54.3	54.4	0.1
Prescription drug use in previous year						
ACE-inhibitor or ARB	96 095 (68.1)	37 734 (64.7)	7.2	67.6	66.0	3.6
ARNI or ivabradin	678 (0.5)	422 (0.7)	3.2	0.6	0.5	1.4
Calcium channel blocker	46 964 (33.3)	17 243 (29.6)	8.0	32.8	30.6	4.8
Loop diuretic	16 390 (11.6)	6896 (11.8)	0.6	11.8	11.5	0.9
Mineralocorticoid receptor antagonist	8498 (6.0)	3171 (5.4)	2.5	6.1	5.5	2.5
Other diuretic	14 739 (10.4)	5900 (10.1)	1.1	10.4	10.3	0.5
Beta-blocker	54 730 (38.8)	18 921 (32.5)	13.3	38.0	34.3	7.8
Digoxin	2867 (2.0)	1097 (1.9)	1.1	2.0	2.0	0.3
Nitrates	12 158 (8.6)	3318 (5.7)	11.4	8.1	6.7	5.4
Platelet inhibitor	48 930 (34.7)	18 752 (32.2)	5.4	33.7	34.1	0.7
Anticoagulant	14 125 (10.0)	4710 (8.1)	6.8	9.7	9.0	2.3
Lipid lowering drug	103 833 (73.6)	40 236 (69.0)	10.2	72.5	71.6	1.9
Antidepressant	22 242 (15.8)	8639 (14.8)	2.6	15.7	15.0	1.9
Antipsychotic	4613 (3.3)	2280 (3.9)	3.4	3.3	3.8	2.7
Anxiolytic, hypnotic, or sedative	21 694 (15.4)	8727 (15.0)	1.1	15.5	14.7	2.3
Beta-2 agonist inhalant	17 706 (12.6)	7486 (12.8)	0.9	12.6	12.7	0.5
Anticholinergic inhalant	5430 (3.9)	2249 (3.9)	0.0	3.8	4.0	1.0
Glucocorticoid inhalant	13 100 (9.3)	5365 (9.2)	0.3	9.3	9.1	0.9
Oral glucocorticoid	10 020 (7.1)	4019 (6.9)	0.8	7.1	6.8	1.3
NSAID	27 077 (19.2)	13 768 (23.6)	10.8	19.9	22.0	5.1
Opiates	22 081 (15.7)	10 106 (17.3)	4.5	15.8	17.0	3.2
No.·of prescription drugs in last year						
0–5	34 733 (24.6)	17 354 (29.8)	11.6	24.9	29.2	9.7
6–10	59 994 (42.5)	24 915 (42.7)	0.4	42.3	43.1	1.5
11–15	30 825 (21.9)	10 776 (18.5)	8.4	21.6	19.0	6.5
>15	15 513 (11.0)	5261 (9.0)	6.6	11.1	8.7	8.1

ACE, angiotensin converting enzyme; ARB, angiotensin receptor blocker; ARNI, angiotensin receptor neprilysin inhibitor; COPD, chronic obstructive pulmonary disease; DPP4, dipeptidyl peptidase 4; GLP1, glucagon-like peptide 1; NSAID, non-steroidal anti-inflammatory drug.

a Not included in the propensity score.

### Primary and secondary outcomes

**Figure 2:**
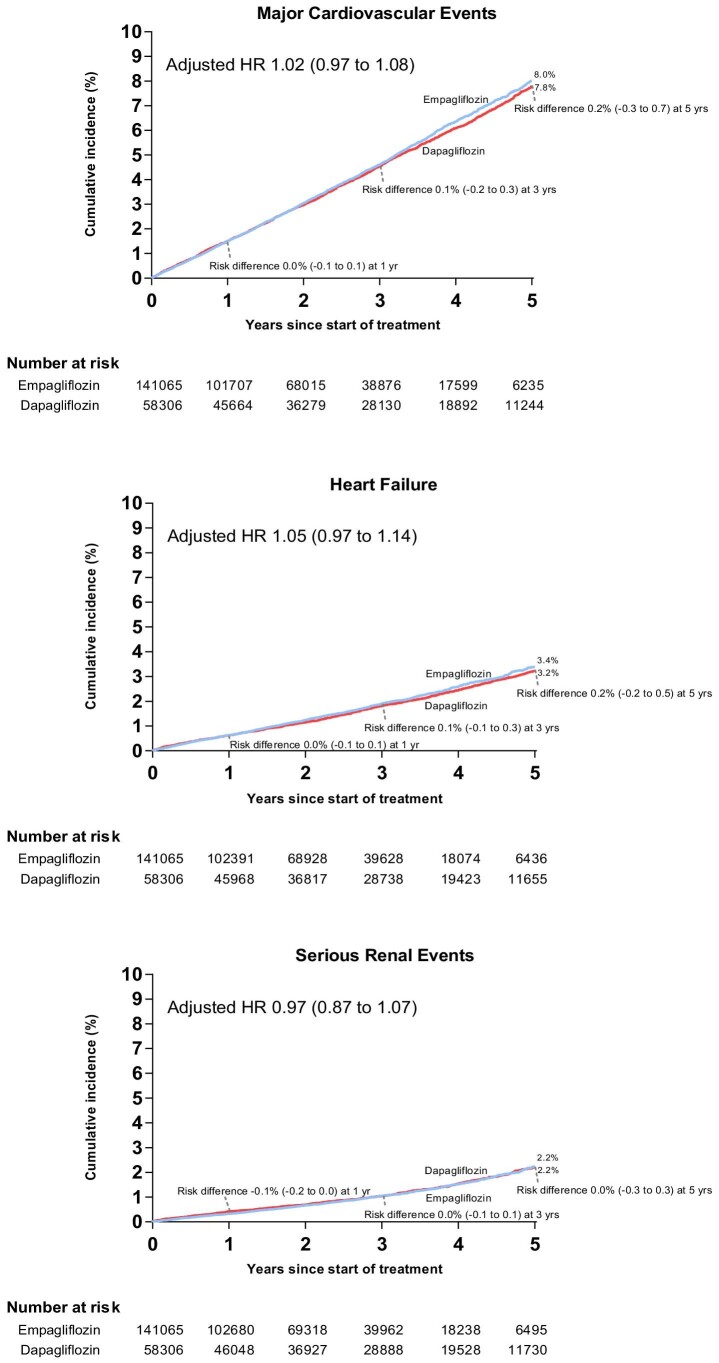
Adjusted cumulative incidence of the coprimary outcomes among users of empagliflozin compared with users of dapagliflozin.

*Table 2* and *Figure 2* show the results of the coprimary and secondary outcome analyses. During follow-up, major cardiovascular events occurred in 4742 users of empagliflozin [adjusted incidence rate (aIR) 15.9 events/1000 person-years] and 2434 users of dapagliflozin (aIR 15.8/1000). Heart failure events occurred in 1901 users of empagliflozin (aIR 6.5/1000) and 1009 users of dapagliflozin (aIR 6.3/1000). Serious renal events occurred in 1101 users of empagliflozin (aIR 3.7 1000) and 652 users of dapagliflozin (aIR 4.0/1000). The adjusted hazard ratio was 1.02 (95% CI 0.97–1.08) for major cardiovascular events, 1.05 (0.97–1.14) for heart failure events and 0.97 (0.87–1.07) for serious renal events.

**Table 2:** Risk of coprimary and secondary outcomes associated with use of empagliflozin, compared with use of dapagliflozin.

	Empagliflozin (*N* = 141 065)		Dapagliflozin (*N* = 58 306)			
Outcomes	No. of events	Adjusted incidence rate (events per 1000 person years)^a^	No. of events	Adjusted incidence rate (events per 1000 person years)^a^	Adjusted hazard ratio (95% CI)^a^	Adjusted rate difference (events per 1000 person years; 95% CI)*^a^*
Coprimary outcomes						
Major cardiovascular events^b^	4742	15.9	2434	15.8	1.02 (0.97 to 1.08)	0.1 (−0.7 to 0.9)
Heart failure^c^	1901	6.5	1009	6.3	1.05 (0.97 to 1.14)	0.2 (−0.3 to 0.6)
Serious renal events^d^	1101	3.7	652	4.1	0.97 (0.87 to 1.07)	−0.4 (−0.8 to 0.0)
Secondary outcomes						
Myocardial infarction	2332	7.7	1221	7.8	1.00 (0.93 to 1.07)	−0.1 (−0.6 to 0.5)
Stroke	1914	6.4	977	6.3	1.03 (0.95 to 1.12)	0.0 (−0.5 to 0.5)
Cardiovascular death	1086	3.6	600	3.8	1.01 (0.92 to 1.13)	−0.2 (−0.6 to 0.2)
Any cause death	4307	14.3	2347	14.5	1.06 (1.00 to 1.11)	−0.3 (−1.0 to 0.5)
Renal replacement therapy	163	0.5	119	0.7	0.77 (0.60 to 0.99)	−0.2 (−0.4 to 0.0)
Death from renal causes	47	0.2	29	0.2	1.20 (0.75 to 1.93)	0.0 (−0.1 to 0.1)
Hospital admission for renal events	994	3.3	570	3.6	1.01 (0.90 to 1.12)	−0.2 (−0.6 to 0.1)
Diabetic ketoacidosis	401	1.4	197	1.2	1.12 (0.94 to 1.33)	0.2 (−0.1 to 0.4)

a Incidence rates, hazard ratios and risk differences adjusted using IPT-weights based on a propensity score that included sociodemographic characteristics, diabetic drug use, co-morbidities, co-medications and health care utilization (Supplementary material online, *Table S5*).

b Defined as composite of myocardial infarction, stroke, and cardiovascular death.

c Defined as hospital admission for, or death due to, heart failure.

d Defined as composite of renal replacement therapy, death from renal causes, and hospital admission for renal events.

Adjusted hazard ratios for the secondary outcomes were 1.00 (95% CI 0.93–1.07) for myocardial infarction, 1.03 (0.95–1.12) for stroke, 1.01 (0.92–1.13) for cardiovascular death, 1.06 (1.00–1.11) for any cause death, 0.77 (0.60–0.99) for renal replacement therapy, 1.20 (0.75–1.93) for renal death and 1.01 (0.90–1.12) for hospitalization for renal events.

Diabetic ketoacidosis occurred in 401 users of empagliflozin (aIR 1.4/1000 person-years) and 197 users of dapagliflozin (1.2/1000). The adjusted hazard ratio was 1.12 (95% CI 0.94–1.33).

### Subgroup analyses

**Figure 3:**
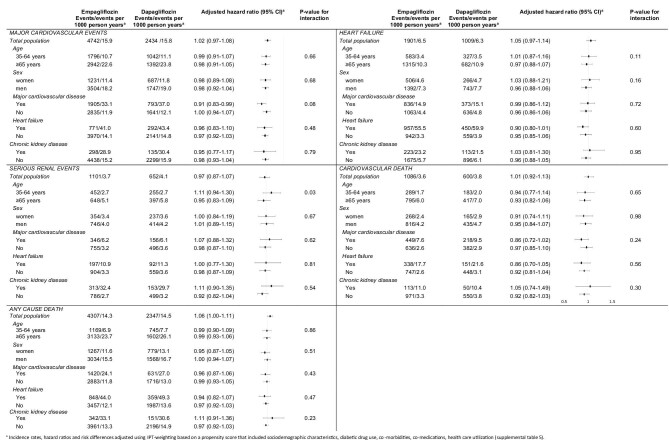
Subgroup analyses of coprimary outcomes and the secondary outcomes cardiovascular death and any cause death among users of empagliflozin compared with users of dapagliflozin.

*Figure 3* shows subgroup analyses. Comparing empagliflozin vs. dapagliflozin for the coprimary outcomes and the secondary outcomes cardiovascular death and any cause death, we observed no significant interactions by subgroup status stratified by sex, history of major cardiovascular disease, history of heart failure, and history and chronic kidney disease. For the coprimary outcome serious renal events we saw a significant interaction by age, the adjusted hazard ratio was 1.11 (95% CI 0.94–1.30) in the age group 35 to <65 years and 0.95 (0.83–1.09) in the age group ≥65 years (*P*-value for interaction 0.03). Results by country are shown in Supplementary material online, *Table S8*.

### Sensitivity analyses

In the sensitivity analyses of the coprimary outcomes with additional adjustment for glycated haemoglobin, blood pressure, albuminuria, eGFR, BMI, and smoking in the Swedish part of the cohort (patient characteristics are shown in Supplementary material online, *Table S9*) and for glycated haemoglobin level, albuminuria, and eGFR in the Danish part of the cohort (patient characteristics are shown in Supplementary material online, *Table S10*) the point estimates for the hazard ratios were largely similar to those of the country-specific analyses without such adjustment (Supplementary material online, *Table S11*), although the point estimate for serious renal events moved towards a slightly higher risk for empagliflozin vs. dapagliflozin. The findings remained consistent for the coprimary outcomes and the secondary outcomes cardiovascular death and any cause death when an as-treated exposure definition was applied (Supplementary material online, *Table S12*).

## Discussion

In this Scandinavian cohort study of patients from routine clinical practice, we observed no statistically significant differences in the risk of major cardiovascular events, heart failure, or serious renal events between empagliflozin and dapagliflozin users. Given the limits of the CIs, the findings of this study were consistent with a relative difference in risk of less than 8% for major cardiovascular events, less than 14% for heart failure and less than 13% for serious renal events, and an absolute difference of less than 1 event per 1000 patient-years for each of these outcomes. This study indicates that there may be no meaningful difference in the cardiorenal effects of empagliflozin and dapagliflozin in routine clinical practice. Furthermore, the risk of diabetic ketoacidosis was similar in users of empagliflozin and users of dapagliflozin. The results for the coprimary outcomes and the secondary outcomes cardiovascular death and any cause death were consistent in subgroups of patients with and without history of major cardiovascular disease, with and without history of heart failure and with and without history of chronic kidney disease. Given the lack of head-to-head data from randomized controlled trials and large and adequately designed observational studies, our study expands substantially on the available evidence of the comparative cardiorenal effectiveness and safety of empagliflozin and dapagliflozin in patients with type 2 diabetes.

A few observational studies have assessed cardiovascular risks associated with empagliflozin as compared to dapagliflozin and have yielded partly inconsistent results.^17,18^ However, the studies were small and limited by a modest number of events, resulting in imprecise estimates. Furthermore, patients with a history of cardiovascular disease or chronic kidney disease were excluded, limiting the generalizability of the findings and both studies suffered from methodological limitations including limited confounding control and potential outcome misclassification. Two observational studies have assessed renal risks associated with empagliflozin in comparison to dapagliflozin and indicate similar effectiveness, but the studies were small and suffered from substantial limitations, including lack of a new user study design.^19,20^

In a Canadian observational study including patients with type 2 diabetes, the adjusted hazard ratio for diabetic ketoacidosis vs. DPP4 inhibitor users were 2.52 (95% CI 1.23–5.14) for users of empagliflozin and 1.86 (1.11–3.10) for users of dapagliflozin. The study suggested that empagliflozin and dapagliflozin were associated with a similar increase in diabetic ketoacidosis risk but was not powered to rule out meaningful clinical differences in risk between the individual drugs. Our neutral findings are inconsistent with a difference in the relative risk of diabetic ketoacidosis associated with use of empagliflozin in comparison to dapagliflozin by more than 31%.

This cohort study of almost 200 000 patients was powered to detect even small differences between empagliflozin and dapagliflozin in risk of cardiovascular and renal outcomes, mortality, and diabetic ketoacidosis. The use of nationwide registers in three countries and the inclusion of a broad study population make the study results generalizable to adults with type 2 diabetes in routine clinical care. Other strengths of this study are the active-comparator new-user design and the use of a propensity score model that included a wide range of patient characteristics to mitigate confounding. In addition, with further adjustment for glycated haemoglobin level, albuminuria, and estimated glomerular filtration rate (Sweden and Denmark), as well as blood pressure, body mass index, and smoking (Sweden) in the Swedish and Danish parts cohort (85.6% of the overall cohort) the results remained consistent which further strengthens the robustness of the findings.

Our study has several limitations. First, the definition of exposure was based on filled prescriptions; low patient adherence may bias the results towards the null. Second, high validity has been observed for procedure codes and diagnoses in the Scandinavian patient registers and validation studies for the codes used for the cardiovascular outcome definitions have shown positive predictive values between 80 and 98%.^21–23^ However, validation studies in the Scandinavian setting have not been conducted for the specific codes used for the renal outcome definitions used in this study, although a differential outcome misclassification between those being treated with empagliflozin and dapagliflozin is improbable. Third, while our exclusion criteria aimed to exclude patients who were prescribed SGLT2 inhibitors for heart failure or chronic kidney disease but did not have type 2 diabetes, they entailed that the likely small subset of patients who have a history of heart failure or chronic kidney disease and are prescribed dapagliflozin or empagliflozin as their first treatment for type 2 diabetes were not included in the study; this could marginally influence the generalizability of our findings. Fourth, empagliflozin and dapagliflozin are by far the most prescribed SGLT2 inhibitors in the Scandinavian countries. Consequently, assessment of the comparative effectiveness of the other approved SGLT2 inhibitors was not feasible. Lastly, this is an observational study; residual confounding by unmeasured factors cannot be ruled out.

## Conclusion

This large comparative study provides evidence in support of similar effectiveness of empagliflozin and dapagliflozin with respect to cardiovascular and renal outcomes and any cause mortality in patients with type 2 diabetes. Moreover, the risk of diabetic ketoacidosis was similar for users of the two drugs.

## Supplementary Material

pvae045_Supplemental_File

## Notes

**Conflict of interest:** All authors have completed the ICMJE uniform disclosure form at www.icmje.org/coi_disclosure.pdf and have the following declarations. Dr Jonasson is an employee of NordicRWE. Dr Eliasson reports personal fees from Amgen, AstraZeneca, Boehringer Ingelheim, Eli Lilly, Merck Sharp & Dohme, Mundipharma, Navamedic, Novo Nordisk, and RLS Global and grants from Sanofi outside the submitted work. Dr Gudbjörnsdottir reports lecture fees and research grants from AstraZeneca, Boehringer Ingelheim, Eli Lilly, Merck Sharp & Dohme, Novo Nordisk, and Sanofi outside the submitted work. No other potential conflicts of interest relevant to this article were reported.

## Data availability

Data extraction is available upon request and approval by the Swedish, Danish and Norwegian Ethical Review Boards and national health data authorities.
