# Supplementary material for: Comparative cardiovascular and renal effectiveness of empagliflozin and dapagliflozin: Scandinavian cohort study
Source: Eur Heart J Cardiovasc Pharmacother. 2024 Jun 25;10(5):432–43. doi: 10.1093/ehjcvp/pvae045 (PMC11411209; doi:10.1093/ehjcvp/pvae045)
Supplement: pvae045_Supplemental_File [file pvae045_supplemental_file.docx]

**Supplementary Appendix**

Engström et al. Comparative cardiovascular and renal effectiveness of empagliflozin and dapagliflozin: Scandinavian cohort study

| **TABLE OF CONTENTS** | **PAGE** |
| --- | --- |
| **Data sources** | 2 |
| **Supplementary table 1.** ATC-codes and estimated days of supply per unit for empagliflozin and dapagliflozin. | 3 |
| **Supplementary table 2.** ICD10 and procedure codes for exclusion criteria. | 4 |
| **Supplementary table 3.** Coprimary outcomes**.** | 6 |
| **Supplementary table 4.** Secondary outcomes | 8 |
| **Supplementary table 5.** Covariates for propensity score. | 10 |
| **Supplementary table 6.** ICD codes for defining history of major cardiovascular disease, chronic kidney disease and heart failure. | 15 |
| **Supplementary table 7.** Variable definitions for the analyses using data from the National Diabetes Register in Sweden and from Danish Register of Laboratory Results for Research in Denmark. | 16 |
| **Supplementary table 8.** Analyses of coprimary outcomes by country. | 17 |
| **Supplementary table 9.** Distribution of variables from the Swedish National Diabetes Register in the Swedish part of the cohort of empagliflozin and dapagliflozin users. Numbers are shown in %. | 18 |
| **Supplementary table 10.** Distribution of glycated hemoglobin, albuminuria and estimated glomerular filtration rate in the Danish part of the cohort of empagliflozin users and dapagliflozin users. Numbers are shown in %. | 20 |
| **Supplementary table 11.** Analyses of coprimary outcomes by country and sensitivity analyses including additional variables in the Swedish and Danish part of the cohort. | 21 |
| **Supplementary table 12.** Additional analyses of coprimary outcomes and the secondary outcomes cardiovascular death and any cause death using an as-treated exposure definition among users of empagliflozin compared with users of dapagliflozin. | 22 |

**Data sources**

Data on filled prescriptions were obtained from the national prescription registers in Sweden^1^, Denmark^2^ and Norway^3^. The registers contain individual-level data on all drug prescriptions filled at all pharmacies in the country since July 2005, in Sweden, 1995 in Denmark, and 2004 in Norway and include the anatomical therapeutic chemical (ATC) code of the dispensed drug, information about the amount of drug dispensed and the date when the prescription was filled.

The national patient registers comprise individual-level data on outpatient and emergency department visits and inpatient admissions to all hospitals in the country.^4–6^ In the present study, these registers were used to obtain information about history of disease at cohort entry for each patient and the outcome events during the study period by using physician-assigned procedure codes, and diagnoses according to the International Classification of Diseases, tenth revision (ICD10).

The population registers were used to obtain information on age, sex, country of birth, migration status, civil status (Norway) and vital status of individual patients^7–9^ Information about patients’ educational level and civil status were obtained from Statistics Denmark and Statistics Sweden.

The National Diabetes Register includes data on risk factors of cardiovascular disease and diabetes complications among patients with type 1 or type 2 diabetes in Sweden. Data are collected by trained nurses and physicians during patient visits to primary care and outpatient clinics nationwide. At present, over 90% of all patients receiving drugs for diabetes in Sweden are included, with this number having increased during the past years.^10^ For the Swedish part of the cohort, we used this register to obtain data on glycated hemoglobin, blood pressure, albuminuria, estimated glomerular filtration rate (eGFR), body-mass index and smoking.

The Danish Register of Laboratory Results for Research collects nationwide data from routine biomarkers from general practitioners and hospital encounters.^11^ Data collection started between 2013 and 2015 depending on region. From this register, we obtained information about glycated haemoglobin, albuminuria and eGFR for the Danish part of the cohort.

The personal identification number assigned to all inhabitants in the three countries enabled linkage of individual-level information across data sources.

| \| **Supplementary table 1.** ATC-codes and estimated days of supply per unit for empagliflozin and dapagliflozin. \| \| \| \| --- \| --- \| --- \| \| **Category** \| **ATC definition** \| **Estimated days of supply (same definition in all countries)** \| \| **Dapagliflozin** \| A10BK01  A10BD15  A10BD21  A10BD25 \| All units are days per tablet  A10BK01 = 1.0  A10BD15 = 0.5  A10BD21 = 1.0  A10BD25 = 0.5 \| \| **Empagliflozin** \| A10BK03  A10BD19  A10BD20 \| A10BK03 = 1.0  A10BD19 = 1.0  A10BD20 = 0.5 \| |
| --- | --- | --- | --- | --- | --- | --- | --- | --- | --- | --- | --- | --- |

| **Supplementary table 2.** ICD10 and procedure codes for exclusion criteria. | | |
| --- | --- | --- |
| **Category** | **Codes (ICD-10, procedure, or ATC)** | **Data source / Type of diagnosis / type of hospital contact** |
| Initiation of both study drugs at the same day | n.a. | Prescribed drug register |
| Dialysis or renal transplantation at any time before index date^a^ | ICD-10: Z49, Z94.0, Z99.2 Procedure: KAS (KKAS in Denmark)  Additional procedure codes:  Sweden: DR012, DR013, DR014, DR015, DR016, DR023, DR024, DR055, DR056, DR060, DR061  Denmark: (B)JFD, (B)JFZ  Norway: A0093, A0094, JAGD30, JAGD31, JAGD32, JAGD50, JAK10, PHGX00, PHGX05, RXGD05, RXGD20, RXGD25, TJA33 | Patient register, any position, any type of hospital contact |
| End stage illness (severe malnutrition, cachexia, dementia, coma) at any time before index date^a^ | ICD-10: E40-E43, F00-F03, G30, R40.2^b^, R64 ATC: N06D | Patient register, any position, any type of hospital contact; prescribed drug register |
| Drug misuse within last year | ICD-10: F11-F16, F18, F19, R78.1-R78.5^b^ T40b  ATC: N07BC | Patient register, any position, any type of hospital contact; prescribed drug register |
| Major pancreatic disease (chronic pancreatitis [defined by pancreatic enzyme substitution prescription within last year or diagnosis at any time before index date], pancreatic cancer, major pancreatic surgery at any time^a^ before index datea) | ICD-10: C25, K86.0, K86.1  Procedure: JLC, JLE ATC: A09AA02 | Patient register, any position, any type of hospital contact; prescribed drug register |
| No specialist care contact or prescription drug in last year prior to the index date | n.a. | Patient register, prescribed drug register |
| Hospital admission within 30 days before index date | n.a. | Patient register |
| Chronic heart failure at any time before index date and no filled prescription of diabetes drug in the last 6 months | ICD-10: I110, I130, I132, I42, I43, I50, J81  ATC: A10 | Patient register, any position, any type of hospital contact; prescribed drug register |
| Chronic kidney disease at any time before index date and no filled prescription of diabetes drug in the last 6 months | E112, E132, E142, I120, I131, I132, N18, N19 | Patient register, any position, any type of hospital contact; prescribed drug register |
| ^a^ Any time is defined as look-back of 10 years in Sweden and Denmark and 5 years in Norway  ^b^ Code not available in Norwegian dataset | | |

| **Supplementary table 3.**  ICD10 and procedure codes used for coprimary outcome definitions. | | |
| --- | --- | --- |
| **Outcome** | **ICD 10 (if not specified) or procedure code** | **Type of diagnosis / type of hospital contact** |
| Major cardiovascular event | Myocardial infarction: I21, I22  Stroke: I60, I61, I63, I64  Cardiovascular death: I00-I99, R570^a^, R960^a^, R961^a^ | Primary diagnosis / inpatient admission; Cause of death, underlying cause of death |
| Heart failure  (hospitalization for heart failure or death due to heart failure) | I110, I130, I132, I50, J81 | Primary diagnosis / inpatient admission; Cause of death, underlying cause of death |
| Serious renal events | Dialysis or renal transplantation  All countries:  Z49, Z940, Z992  Sweden:  procedure code:  KAS, DR012, DR013, DR014  DR015, DR016, DR023, DR024, DR055, DR056, DR060, DR061  Denmark:  Procedure code: KKAS, BJFD, BJFZ  Norway:  Procedure code:  KAS, A0093, A0094, JAGD30, JAGD31, JAGD32, JAGD50, JAK10, PHGX00, PHGX05, RXGD05, RXGD20, RXGD25, TJA33  Death from renal causes  All countries:  E112, E132, E142, I120, I131, I132  N00-N08, N10-N16, N17, N18, N19,  N20-N23, N25-N29  Hospitalization for renal events  All countries:  E112, E132, E142, I120, I131, I132, N17,  N18, N19 | Any position / inpatient admission or outpatient visit  Cause of death, underlying cause of death  Primary diagnosis/inpatient admission |

|  | | |
| --- | --- | --- |
| **Supplementary table 4.** ICD10 and procedure codes used for secondary outcome definitions | | |
|  | ICD 10 code | Type of diagnosis (primary, secondary) / type of hospital contact |
| **Cardiovascular** |  |  |
| Myocardial infarction | I21, I22 | Primary diagnosis / inpatient admission; Cause of death, underlying cause of death |
| Stroke | I60, I61, I63, I64 | Primary diagnosis / inpatient admission; Cause of death, underlying cause of death |
| Cardiovascular death | I00-I99, R570^a^, R960^a^, R961^a^ | Cause of death, underlying cause of death |
| Any-cause death | n.a. | n.a. |
| **Renal (components of the composite renal outcome)** |  |  |
| Dialysis or renal transplantation | Dialysis or renal transplantation  All countries:  Z49, Z940, Z992  Sweden:  procedure code:  KAS, DR012, DR013, DR014  DR015, DR016, DR023, DR024, DR055, DR056, DR060, DR061  Denmark:  Procedure code: KKAS, BJFD* BJFZ*  Norway:  Procedure code:  KAS, A0093, A0094, JAGD30, JAGD31, JAGD32, JAGD50, JAK10, PHGX00, PHGX05, RXGD05, RXGD20, RXGD25, TJA33 | Any position / inpatient admission or outpatient visit |
| Death from renal causes | E112, E132, E142, I120, I131, I132  N00-N08, N10-N16, N17, N18, N19,  N20-N23, N25-N29 | Cause of death, underlying cause of death |
| Hospitalization for renal events | E112, E132, E142, I120, I131, I132, N17,  N18, N19 | Primary diagnosis/inpatient admission |
| **Adverse events** |  |  |
| Diabetic ketoacidosis | Sweden: E110A, E111, E111A, E131, E141  Denmark: E111, E131, E141  Norway: E111, E131, E141 | Any position / inpatient admission |

| **Supplementary table 5.** Covariates for propensity score. | |
| --- | --- |
| **Sociodemographic characteristics** | **ICD/categories** |
| Sex | Women/men |
| Age | 5-year categories |
| Place of birth | Scandinavia; Rest of Europe; Outside Europe, Missing |
| Living with partner | Yes/no |
| Education ^a^ | Primary school and high school; vocational or short-term tertiary education; medium or long tertiary education; missing; Norway |
| Calendar year^b^ | 2-year category |
| **Medical history (10 yr look-back in Sweden and Denmark; 5 year look-back in Norway)** | *ICD-10 code and procedure code* |
| Acute coronary syndrome | ICD-10: I200, I21-22 |
| Other ischemic heart disease | ICD-10: I11 (not I110), I20 (not I200), I24, I25 |
| Heart failure/cardiomyopathy | ICD-10: I50, I110, I130, I132, I42, I43, J81 |
| Valve disorders | ICD-10: I34-I37 |
| Stroke | ICD-10: I60-I64 |
| Other cerebrovascular disease | ICD-10: I65-I69, G45 (excl G454), G46 |
| Atrial fibrillation | ICD-10: I48 |
| Other arrhythmia | ICD-10: I44-I47, I49 |
| Arterial disease (including amputation) | ICD-10: I65, I70, I72, I73, I74, I77, K550, K551, E115, E145, E135  Procedure: NFQ ,NGQ, NHQ |
| Chronic kidney disease | ICD-10:  E112, E132, E142, I120, I131, I132, N18, N19 |
| Other renal disease | ICD-10:  N00-08, N10-N16, N17, N20-N23, N25-N29 |
| Diabetes complications | ICD-10: E110, E111, E113, E114, E116, E117, E118, E130, E131, E133, E134, E136, E137, E138, E140, E141, E143, E144, E146, E147, E148, E160, E161, E162, G990, G590, G632, H280, H358, H360, M142, M146, M908, L984  Procedure: CKC10, CKC12, CKC15, CKD65 |
| COPD | ICD-10: J44 |
| Other lung disease | ICD-10: I27, J84, R092, E662, Z99, J40-J43, J45-J47, J60-J69, J70,^c^ J92, J96, J982, J983  Procedure: GBB |
| Venous thromboembolism | ICD-10: I26, I80 (except I80.0), I81, I820, I822-I829 |
| Cancer (excl non-melanoma skin cancer) | ICD-10: C00-C43, C45-C97 |
| Liver disease | ICD-10: B18, I850, I859, I982, K70-K77 |
| Rheumatic disease | ICD-10: M05-M09, M30-34, M351, M353, M45 |
| Psychiatric disorder | ICD-10: F04-F09, F20-F99 |
| Alcohol related disorders | ICD10: F10. ATC: N07BB, |
| **Medical history (1 yr look-back)** | *ICD-10 code and procedure code* |
| Coronary revascularization in previous yr | Procedure: FNA, FNB, FNC, FND, FNE, FNG, FNP02, FNP12, FNQ05, FNQ12, FNR22 |
| Other cardiac surgery/invasive cardiac procedure in previous yr | Procedure: F (except FNA, FNB, FNC, FND, FNE, FNG, FNP02, FNP12, FNQ05, FNQ12, FNR22, FPFE, FPGX), DF020 |
| Fracture in previous year | ICD-10: S02 (except S025), S12, S22, S32, S42, S52, S62, S72, S82, S92, T02, T08, T10, T12, M484, M485, M843 |
| **Prescription-drug use in previous yr** | *ATC code* |
| ACE-inhibitor or ARB | C09A-D (except C09DX04) |
| ARNI or ivabradin | C09DX04, C01EB17 |
| Calcium channel blocker | C08C, C08D |
| Loop diuretic | C03C, C03EB |
| Mineralocorticoid receptor antagonist | C03DA |
| Other diuretic | C03A, C03B, C03EA |
| Beta-blocker | C07 |
| Digoxin | C01AA05 |
| Nitrates | C01DA |
| Platelet inhibitor | B01AC |
| Anticoagulant | B01AA, B01AE07, B01AF, B01AX05 |
| Lipid lowering drug | C10 |
| Antidepressant | N06A |
| Antipsychotic | N05A |
| Anxiolytic, hypnotic or sedative | N05B, N05C |
| Beta-2 agonist inhalant | R03AC, R03AK, R03AL |
| Anticholinergic inhalant | R03BB, R03AL |
| Glucocorticoid inhalant | R03BA, R03AK, R03AL08, R03AL09, R03AL11, R03AL12 |
| Oral glucocorticoid | H02AB |
| NSAID | M01A |
| Opiates | N02A |
| **Diabetes drugs in the last 6 months** |  |
| No diabetes drug | Not any A10 |
| Metformin | A10BA02, A10BD02, A10BD03, A10BD05, A10BD07, A10BD08, A10BD10, A10BD11, A10BD13, A10BD14, A10BD15, A10BD16, A10BD20 |
| Sulfonylureas | A10BB, A10BD01, A10BD02, A10BD04, A10BD06 |
| GLP1RA | A10BJ01, A10BJ02, A10BJ03, A10BJ05, A10BJ06, A10AE54, A10AE56 (not including saxenda -product no. 131577, 395175 164108, 439932 or 575140 in Denmark and Norway, 034982 in Norway and 513490, 141823, 439932, 026334 or 471462 in Sweden), |
| DPP4 inhibitors | A10BH01, A10BH02, A10BH03, A10BH04, A10BH05, A10BD07, A10BD08, A10BD09, A10BD10, A10BD11, A10BD13, A10BD19, A10BD21, A10BD24, A10BD25 |
| Insulin | A10AB, A10AC, A10AD, A10AE |
| Other antidiabetics (glitazones, glinides, acarbose) | A10BF01, A10BG, A10BD03, A10BD04, A10BD05, A10BD06, A10BD09, A10BD14, A10BX, |
| Time since first diabetes drug | A10;  <1 year, 1-3 years, ≥3 to 5 years, ≥5 to 7 years, ≥7 years |
| **Health care utilization in previous year** |  |
| No. of drugs used ^c^ | <5, 6-10, 11-15, >15 |
| Hospitalization due to cardiovascular causes | I00-I99 (primary position) |
| Hospitalization due to heart failure | I110, I130, I132, I42, I43, I50, J81 (primary position) |
| Hospitalization due to renal causes | E112, E132, E142, I120, I131, I132  N00-N08, N10-N16, N17, N18, N19,  N20-N23, N25-N29 (primary position) |
| Hospitalization due to type 2 diabetes | E11 (primary position) |
| Hospitalization due to other | Not I00-I99, E11, N00-29 (primary position) |
| Outpatient contact due to cardiovascular causes | I00-I99 (primary position) |
| Outpatient contact due to heart failure | I110, I130, I132, I42, I43, I50, J81 (primary position) |
| Outpatient contact due to renal causes | E112, E132, E142, I120, I131, I132  N00-N08, N10-N16, N17, N18, N19,  N20-N23, N25-N29 (primary position |
| Outpatient contact due to type 2 diabetes | E11 (primary position) |
| Outpatient contact due to other causes | Not I00-I99, E11, N00-29 (primary position) |
| Abbreviations: ACE, angiotensin converting enzyme; ARB, angiotensin receptor blocker; ARNI, angiotensin receptor neprilysin inhibitor; COPD, chronic obstructive pulmonary disease; DPP4, dipeptidyl peptidase 4; GLP1, glucagon-like peptide 1; NSAID, non-steroidal anti-inflammatory drug.  ^a^ Information about education is not available in Norway.  ^b^ Not included in the propensity score  ^c^ In the Norwegian dataset, the number of drugs used for the ATC-codes included as variables in the propensity score were used. In Sweden and Denmark, all ATC-codes were used. | |

| **Supplementary table 6.** ICD codes for defining history of major cardiovascular disease, chronic kidney disease and heart failure. | | |
| --- | --- | --- |
|  | **ICD 10/procedure code** | **Type of diagnosis / hospital contact** |
| Major cardiovascular disease | Coronary revascularization: FNA, FNB, FNC, FND, FNE, FNG, FNP02, FNP12, FNQ05, FNQ12, FNR22  Acute coronary syndrome: I200, I21-22  Stroke: I60-I64  Peripheral arterial disease: I65, I70, I72, I73.9, K550, K551, E115, E135, E145 | Any/any |
| Heart failure | I110, I130, I132, I42, I43, I50, J81 | Any/any |
| Chronic kidney disease | E112, E132, E142, I120, I131, I132, N18, N19 | Any/any |
| 10 year look-back in Sweden and Denmark; 5 year look-back in Norway | | |

| **Supplementary table 7.** Variable definitions for the analyses using data from the National Diabetes Register in Sweden and from Danish Register of Laboratory Results for Research in Denmark. | | | |
| --- | --- | --- | --- |
| **Variable** | **Categorization** | **% missing values (Sweden)^a^** | **% missing values (Denmark)^b^** |
| HbA1c (mmol/mol) | ≤52; 53-62; 63-72; 73-82; ≥83 | 44.0 | 18.2 |
| Albuminuria | Normalbuminuria; microalbuminuria; macroalbuminuria | 36.3 | 29.2 |
| eGFR (ml/min) | <30; 30-59; 60-89; ≥90 | 25.5 | 7.3 |
| Blood pressure | *Normotension:*  SBP <140 mmHg AND DBP <90 mmHg  *Stage 1 hypertension:*  SBP ≥140 to <160 mmHg OR DBP: ≥90 to <100mmHg  *Stage 2 hypertension:*  SBP ≥160 mmHg OR DBP: ≥100 mmHg | 22.0 | - |
| Body-mass index (kg/m2) | Normal weight: <25  Overweight: ≥25 to <30  Obese class I: ≥30 to <35  Obese class II: ≥35 | 27.8 | - |
| Current smoking | Yes/no | 28.0 | - |

Abbreviations: SBP: systolic blood pressure; DBP: diastolic blood pressure; eGFR: estimated glomerular filtration rate.

a Missing values in the Swedish part of the cohort of empagliflozin users and dapagliflozin users.

b Missing values in the Danish part of the cohort of empagliflozin users and dapagliflozin users.

| **Supplementary table 8.** Analyses of coprimary outcomes by country. | | | | | | |
| --- | --- | --- | --- | --- | --- | --- |
|  | **Empagliflozin** | | **Dapagliflozin** | |  | |
|  | **No. of patients** | **Events/events per 1000 person years^a^** | **No. of patients** | **Events/events per 1000 person years^a^** | **Adjusted HR (95% CI) ^a^** | |
| **Sweden** |  |  |  |  |  |  |
| Major cardiovascular events^b^ | 86 336 | 3088/17.3 | 20 036 | 1068/18.4 | 0.97 (0.90-1.04) |  |
| Heart failurc^e^ | 86 336 | 1112/6.4 | 20 036 | 410/6.9 | 0.95 (0.84-1.08) |  |
| Serious renal eventd^f^ | 86 336 | 461/2.6 | 20 036 | 176/2.7 | 1.08 (0.90-1.31) |  |
| **Denmark** |  |  |  |  |  |  |
| Major cardiovascular events^b^ | 40 543 | 1299/13.3 | 23 775 | 781/12.5 | 1.08 (0.99-1.19) |  |
| Heart failure^c^ | 40 543 | 704/7.4 | 23 775 | 445/6.6 | 1.13 (1.00-1.27) |  |
| Serious renal events^d^ | 40 543 | 584/6.0 | 23 775 | 347/5.3 | 1.13 (0.99-1.30) |  |
| **Norway** |  |  |  |  |  |  |
| Major cardiovascular events^b^ | 14 183 | 355 (16.4) | 14 490 | 585 (17.5) | 0.94 (0.82-1.09) |  |
| Heart failure^c^ | 14 183 | 85 (3.5) | 14 490 | 154 (5.0) | 0.83 (0.62-1.11) |  |
| Serious renal events^d^ | 14183 | 56 (2.5) | 14490 | 129 (3.9) | 0.71 (0.51-1.00) |  |

^a^ Adjusted using IPT-weighting based on a propensity score that included sociodemographic characteristics, diabetic drug use, co-morbidities, co-medications and health care utilization (Table 1).

^b^ Defined as composite of myocardial infarction, stroke, and cardiovascular death

^c^ Defined as hospital admission for, or death due to, heart failure.

^d^ Defined as composite of renal replacement therapy, death from renal causes, and hospital admission for renal events

| **Supplementary table 9.** Distribution of variables from the Swedish National Diabetes Register in the Swedish part of the cohort of empagliflozin and dapagliflozin users. Numbers are shown in %. | | |
| --- | --- | --- |
|  | **Empagliflozin**  **(N=86336)** | **Dapagliflozin**  **(N=20036)** |
| **Blood pressure (mmHg)** | 40089 (46.4) | 8282 (41.3) |
| SBP < 140 and DBP < 90 | 24457 (28.3) | 5513 (27.5) |
| SBP 140-159 or DBP 90-99 | 3752 (4.3) | 869 (4.3) |
| SBP ≥160 or DBP ≥100 | 18038 (20.9) | 5372 (26.8) |
| Missing |  |  |
| **HbA1c (mmol/mol)** | 7009 (8.1) | 1206 (6.0) |
| ≤52 | 15375 (17.8) | 2635 (13.2) |
| 53-62 | 12394 (14.4) | 2666 (13.3) |
| 63-72 | 7345 (8.5) | 1768 (8.8) |
| 73-82 | 7325 (8.5) | 1829 (9.1) |
| ≥83 | 36888 (42.7) | 9932 (49.6) |
| Missing |  |  |
| **Body mass index (kg/m2)** | 6326 (7.3) | 1150 (5.7) |
| <25 | 21981 (25.5) | 4383 (21.9) |
| 25-29 | 20806 (24.1) | 4695 (23.4) |
| 30-34 | 14106 (16.3) | 3386 (16.9) |
| ≥35 | 23117 (26.8) | 6422 (32.1) |
| Missing |  |  |
| **Albuminuria** | 42913 (49.7) | 8461 (42.2) |
| Normalbuminuria | 11010 (12.8) | 2439 (12.2) |
| Microalbuminuria | 2283 (2.6) | 604 (3.0) |
| Macroalbuminuria | 30130 (34.9) | 8532 (42.6) |
| Missing |  |  |
| **eGFR (ml/min)** | 30018 (34.8) | 6584 (32.9) |
| ≥90 | 29537 (34.2) | 5952 (29.7) |
| 60-89 | 5823 (6.7) | 1268 (6.3) |
| 30-59 | 70 (0.1) | 30 (0.1) |
| <30 | 20888 (24.2) | 6202 (31.0) |
| Missing |  |  |
| **Current smoking** | 8912 (10.3) | 1875 (9.4) |
| No | 54449 (63.1) | 11348 (56.6) |
| Yes | 22975 (26.6) | 6813 (34.0) |
| Missing | 40089 (46.4) | 8282 (41.3) |
| Abbreviations: SBP, systolic blood pressure; DBP, diastolic blood pressure; eGFR, estimated glomerular filtration rate. | | |

| **Supplementary table 10.** Distribution of glycated hemoglobin, albuminuria and estimated glomerular filtration rate in the Danish part of the cohort of empagliflozin users and dapagliflozin users. Numbers are shown in %. | | |  |
| --- | --- | --- | --- |
|  | **Empagliflozin**  **(N=40543)** | **Dapagliflozin**  **(N=23775)** | |
| **HbA1c (mmol/mol)** |  |  | |
| ≤52 | 3321 (8.2) | 2177 (9.2) | |
| 53-62 | 10301 (25.4) | 6024 (25.3) | |
| 63-72 | 8628 (21.3) | 4910 (20.7) | |
| 73-82 | 5126 (12.6) | 2950 (12.4) | |
| ≥83 | 5868 (14.5) | 3289 (13.8) | |
| Missing | 7299 (18.0) | 4425 (18.6) | |
| **Albuminuria** |  |  | |
| Normalbuminuria | 20333 (50.2) | 10907 (45.9) | |
| Microalbuminuria | 7835 (19.3) | 4057 (17.1) | |
| Macroalbuminuria | 1529 (3.8) | 906 (3.8) | |
| Missing | 10846 (26.8) | 7905 (33.2) | |
| **eGFR (ml/min)** |  |  | |
| ≥90 | 19144 (47.2) | 11197 (47.1) | |
| 60-89 | 14928 (36.8) | 8503 (35.8) | |
| 30-59 | 3534 (8.7) | 2127 (8.9) | |
| <30 | 94 (0.2) | 112 (0.5) | |
| Missing | 2843 (7.0) | 1836 (7.7) | |
| Abbreviations: SBP, systolic blood pressure; DBP, diastolic blood pressure; eGFR, estimated glomerular filtration rate. | | | |

| **Supplementary table 11.** Analyses of coprimary outcomes by country and sensitivity analyses including additional variables in the Swedish and Danish part of the cohort. | | | | | | | |
| --- | --- | --- | --- | --- | --- | --- | --- |
|  | **Empagliflozin** | | **Dapagliflozin** | |  | |  |
|  | **No. of patients** | **Events/events per 1000 person years^a^** | **No. of patients** | **Events/events per 1000 person years^a^** | **Adjusted HR (95% CI) ^a^** | | **Adjusted for additional variables**  **HR (95% CI)^b^** |
| **Sweden** |  |  |  |  |  |  | |
| Major cardiovascular events^d^ | 86 336 | 3088/17.3 | 20 036 | 1068/18.4 | 0.97 (0.90-1.04) | 0.99 (0.92-1.06) | |
| Heart failure^e^ | 86 336 | 1112/6.4 | 20 036 | 410/6.9 | 0.95 (0.84-1.08) | 0.99 (0.87-1.11) | |
| Serious renal events^f^ | 86 336 | 461/2.6 | 20 036 | 176/2.7 | 1.08 (0.90-1.31) | 1.15 (0.96-1.39) | |
| **Denmark** |  |  |  |  |  |  | |
| Major cardiovascular events^b^ | 40 543 | 1299/13.2 | 23 775 | 781/12.5 | 1.08 (0.99-1.19) | 1.08 (0.99-1.19) | |
| Heart failure^c^ | 40 543 | 704/7.3 | 23 775 | 445/6.6 | 1.13 (1.00-1.27) | 1.14 (1.00-1.28) | |
| Serious renal events^d^ | 40 543 | 584/5.9 | 23 775 | 347/5.5 | 1.13 (0.99-1.30) | 1.19 (1.03-1.36) | |

^a^ Adjusted using IPT-weighting based on a propensity score that included sociodemographic characteristics, diabetic drug use, co-morbidities, co-medications and health care utilization (Table 1).

^b^In addition to the variables presented in Table 1, these analyses used a propensity score including additional variables, including glycated hemoglobin, blood pressure, albuminuria, estimated glomerular filtration rate, body mass index, and smoking

^c^In addition to the variables presented in Table 1, these analyses used a propensity score including additional variables, including glycated hemoglobin, albuminuria, and estimated glomerular filtration rate

^d^ Defined as composite of myocardial infarction, stroke, and cardiovascular death

^e^ Defined as hospital admission for, or death due to, heart failure.

^f^ Defined as composite of renal replacement therapy, death from renal causes, and hospital admission for renal events.

| **Supplementary table 12.** Additional analyses of coprimary outcomes and the secondary outcomes cardiovascular death and any cause death using an as-treated exposure definition among users of empagliflozin compared with users of dapagliflozin. | | | | | | |
| --- | --- | --- | --- | --- | --- | --- |
|  | **Empagliflozin** | | **Dapagliflozin** | |  | |
|  | **Events** | **Adjusted incidence rate (events per 1000 person- years)** | **Events** | **Adjusted incidence rate (events per 1000 person- years)** | **Adjusted HR (95% CI) ^a^** | |
| **Coprimary outcomes** |  |  |  |  |  |  |
| Major cardiovascular events^b^ | 1900 | 13.9 | 771 | 13.7 | 1.01 (0.93-1.10) |  |
| Heart failure^c^ | 720 | 5.5 | 334 | 5.5 | 0.99 (0.87-1.14) |  |
| Serious renal events^d^ | 397 | 2.9 | 181 | 3.2 | 0.91 (0.76-1.09) |  |
| **Secondary outcomes** |  |  |  |  |  |  |
| Cardiovascular death | 363 | 2.6 | 152 | 2.6 | 1.01 (0.83-1.23) |  |
| Any cause death | 1282 | 9.4 | 556 | 9.6 | 0.99 (0.89-1.09) |  |

^a^ Adjusted using IPT-weighting based on a propensity score that included sociodemographic characteristics, diabetic drug use, co-morbidities, co-medications and health care utilization (Table 1)

^b^ Defined as composite of myocardial infarction, stroke, and cardiovascular death

^c^ Defined as hospital admission for, or death due to, heart failure.

^d^ Defined as composite of renal replacement therapy, death from renal causes, and hospital admission for renal events.

**References**

1. Wettermark B, Hammar N, MichaelFored C, Leimanis A, Olausson PO, Bergman U, et al. The new Swedish Prescribed Drug Register—Opportunities for pharmacoepidemiological research and experience from the first six months. *Pharmacoepidemiology and Drug Safety*. 2007;16(7):726–735.

2. Pottegård A, Schmidt SAJ, Wallach-Kildemoes H, Sørensen HT, Hallas J, Schmidt M. Data Resource Profile: The Danish National Prescription Registry. *International Journal of Epidemiology.* 2017;46(3):798–798f.

3. Norwegian Prescription Database - NIPH n.d. https://www.fhi.no/en/hn/health-registries/norpd/norwegian-prescription-database/ (accessed February 16, 2023).

4. Ludvigsson JF, Andersson E, Ekbom A, Feychting M, Kim J-L, Reuterwall C, et al. External review and validation of the Swedish national inpatient register. *BMC Public Health*. 2011;11:450.

5. Schmidt M, Schmidt SAJ, Sandegaard JL, Ehrenstein V, Pedersen L, Sørensen HT. The Danish National Patient Registry: a review of content, data quality, and research potential. *Clinical Epidemiology*. 2015;7:449–490.

6. Overview of the national health registries - NIPH n.d. https://www.fhi.no/en/more/access-to-data/about-the-national-health-registries2/ (accessed February 16, 2023).

7. Ludvigsson JF, Almqvist C, Bonamy A-KE, Ljung R, Michaëlsson K, Neovius M, et al. Registers of the Swedish total population and their use in medical research. *European Journal of Epidemiology*. 2016;31(2):125–136.

8. Schmidt M, Pedersen L, Sørensen HT. The Danish Civil Registration System as a tool in epidemiology. *European Journal of Epidemiology*. 2014; 29:541–549.

9. National Population Register - The Norwegian Tax Administration n.d. https://www.skatteetaten.no/en/person/national-registry/ (accessed February 16, 2023).

10. Eeg-Olofsson K, Åkesson K, Nåtman J et al. National Diabetes Register. Annual Report 2021. https://www.ndr.nu/pdfs/Arsrapport_NDR_2021.pdf (accessed February 16, 2023).

11. Arendt JFH, Hansen AT, Ladefoged SA, Sørensen HT, Pedersen L, Adelborg K. Existing Data Sources in Clinical Epidemiology: Laboratory Information System Databases in Denmark. *Clinical Epidemiology*. 2020;12:469-475.
